# Supplementary material for: The chromatin remodelers RSC and ISW1 display functional and chromatin-based promoter antagonism
Source: eLife. 2015 Mar 30;4:e06073. doi: 10.7554/eLife.06073 (PMC4423118; doi:10.7554/eLife.06073)
Supplement: Supplementary file 1. — Table of yeast strains. List of yeast strains and their genotypes used in this study. DOI: http://dx.doi.org/10.7554/eLife.06073.019 [file elife06073s001.docx]

# Supplemental Table 1. Yeast Strains

| **Strain** | **Mating Type** | **Genotype** |
| --- | --- | --- |
| YBC26 | *MATa* | *his4-912∂ lys2-128∂ leu2∆1 ura3-52 snf2∆::LEU2* |
| YBC62 | *MATa* | *lys2-128∂ leu2∆1 ura3-52 trp1∆63 his3∆200* |
| YBC601 | *MATa* | *ura3-52* |
| YBC627 | *MATα* | *lys2-128∂ leu2∆1 ura3-52 trp1∆63 his3∆200 rsc4∆::HIS3 [p164; pRS316.RSC4]* |
| YBC803 | *MATa* | *his3Δ200 leu2Δ1 lys2Δ0 trp1Δ63 ura3-52 rsc1Δ::HIS3 rsc2Δ::LEU2 [p316.RSC1]* |
| YBC906 | *MATa* | *lys2-128∂ leu2∆1 ura3-52 trp1∆63 his3∆200 rsc3∆::HIS3 [315.rsc3-3]* |
| YBC1111 | *MATa* | *his3Δ200 leu2Δ1 lys2Δ0 trp1Δ63 ura3-52 rsc1Δ::HIS3 rsc2Δ::LEU2 [p314.rsc2.V457M]* |
| YBC1231 | *MATa* | *his3Δ200 leu2Δ1 lys2Δ0 trp1Δ63 ura3-52 rsc1Δ::HIS3 rsc2Δ::LEU2 [p314.RSC2]* |
| YBC1245 | *MATa* | *his3∆200 leu2∆0 (or ∆1) lys2∆0 (or 128∂) trp1∆63 ura3-52 (or ∆0) rsc1∆::LEU2 rsc2∆::LEU2 set1∆::HIS3MX6 [p316.RSC1]* |
| YBC1278 | *MATα* | *lys2-128∂ leu2∆1 ura3-52 trp1∆63 his3∆200 rsc4∆::HIS3 [p1083; pRS314 - rsc4-2]* |
| YBC1333 | *MATα* | *his3∆200 leu2∆1 trp1∆63 ura3-52 lys2-128∂ rsc7∆::HIS3 [p137;RSC7.URA3]* |
| YBC1416 | *MATa* | *lys2-128∂ leu2∆1 ura3-52 trp1∆63 his3∆200 isw1∆::KANMX* |
| YBC1479 | *MATa* | *his3∆200 leu2∆1 lys2∆0 trp1∆63 ura3∆0(or -52) rsc1∆::HIS3 rsc2∆::LEU2 isw1∆::kanmx [316.RSC1]* |
| YBC1480 | *MATa* | *his3∆200 leu2∆1 lys2∆0 trp1∆63 ura3∆0(or -52) rsc1∆::HIS3 rsc2∆::LEU2 isw2∆::kanmx [316.RSC1]* |
| YBC1485 | *MATα* | *lys2-128∂ leu2∆1 ura3-52 trp1∆63 his3∆200 rsc3∆::HIS3 isw1∆::KANMX [p316.RSC3]* |
| YBC1683 | *MATa* | *leu2∆0 (or ∆1) lys2∆0 (or -128∂) ura3∆0 (or -52) his3∆1(or ∆200) trp1∆63 rsc1∆::HIS3 rsc2∆::LEU2 dot1∆::kanmx [316.RSC1]* |
| YBC1894 | *MATa* | *his3∆1 leu2∆0 met15∆0 ura3∆0* |
| YBC2039 | *MATa* | *his3∆200 leu2∆1 lys2-128∂ trp1∆63 ura3-52 rsc7∆::LEU2 can1∆::MFA1pr-HIS3 [p137;RSC7.URA3]* |
| YBC2140 | *MATa* | *hht1-hhf1::pWZ405-F2F9-LEU2, hht2-hhf2::pWZ403-F4F10-HIS3 rsc1∆::HIS3 rsc2.V457M ura3-52(or ∆0) lys2-801(or ∆0) ade2-101(or ade2∆::hisG) trp1∆63 his3∆200 leu2∆1(or ∆0) met15∆0 [H3-H4 WT.URA3]* |
| YBC2191 | *MATa* | *ura3-52 trp1∆63 his3∆200 leu2::PET56 GAL1prom-UBR1::HIS3 sth1::CUP1prom-1xHA-STH1td::URA3* |
| YBC2192 | *MATa* | *ura3-52 trp1∆63 his3∆200 leu2::PET56 ubr1Δ::HIS3 sth1::CUP1prom-1xHA-STH1td::URA3* |
| YBC2233 | *MATa* | *lys2-128∂ leu2∆1(or ∆0) ura3-52 trp1∆63 his3∆200 isw1∆::KanMX rsc7∆::HIS3[316.RSC7]* |
| YBC2467 | *MATα* | *ura3-52 his3Δ200 trp1Δ63 leu2 lys2−128δ isw1Δ::KanMX GAL1prom-UBR1::HIS3 sth1::CUP1prom-1xHA-STH1td::URA3* |
| YBC2468 | *MATα* | *ura3-52 his3Δ200 trp1Δ63 leu2 lys2−128δ isw1Δ::KanMX ubr1Δ::HIS3 sth1::CUP1prom-1xHA-STH1td::URA3* |
| YBC2693 | *MATa* | *his3∆1 leu2∆0 met15∆0 ura3∆0 ioc3∆::Kanmx* |
| YBC2729 | *MATa* | *his3∆1(or ∆200) leu2∆0(or ∆1) trp1∆63 ura3∆0(or -52) rsc1∆::HIS3 rsc2∆::LEU2 ioc2∆::KanMX [316.RSC2]* |
| YBC2730 | *MATα* | *his3∆1(or ∆200) leu2∆0(or ∆1) trp1∆63 ura3∆0(or -52) rsc1∆::HIS3 rsc2∆::LEU2 ioc3∆::KanMX [316.RSC2]* |
| YBC2731 | *MATa* | *his3∆1(or ∆200) leu2∆0(or ∆1) trp1∆63 ura3∆0(or -52) met15∆0 ade2∆::hisG rsc1∆::HIS3 rsc2∆::LEU2 ioc4∆::KanMX [316.RSC2]* |
| YBC2744 | *MATa* | *his3∆200 leu2∆0 (or ∆1) lys2∆0 (or -128∂) trp1∆63 ura3-52 (or ∆0) rsc1∆::LEU2 rsc2∆::HIS3 set1∆::HIS3MX6 isw1∆::kanmx [316.RSC1]* |
| YBC2803 | *MATa* | *his3∆200(or ∆1) leu2∆0 (or ∆1) lys2∆0 (or -128∂) trp1∆63 ura3-52 (or ∆0) rsc1∆::LEU2 rsc2∆::HIS3 set1∆::HIS3MX6 ioc3∆::kanmx[316.RSC1]* |
| YBC2810 | *MATa* | *his3∆200 leu2∆1 lys2∆0 trp1∆63 ura3∆0(or -52) rsc1∆::HIS3 rsc2∆::LEU2 isw1∆::kanmx* |
| YBC2812 | *MATa* | *lys2-128∂ leu2∆1 ura3-52 his3∆200 his4-912∂ snf2∆::LEU2 isw1∆::KANMX* |
| YBC2867 | *MATα* | *lys2-128∂ leu2∆1 ura3-52 trp1∆63 his3∆200 rsc4∆::HIS3 isw1∆::kanmx* |
| YBC2882 | *MATa* | *ura3-52 RSC8.9XMYC::TRP1* |
| YBC2883 | *MATa* | *ura3-52 IOC3.13XMYC::TRP1* |
| YBC2942 | *MATa* | *lys2-128∂ leu2∆1 ura3-52 trp1∆63 his3∆200 rsc4∆::HIS3 isw1∆::kanmx [316.RSC4]* |
| YBC3010 | *MATa* | *ura3-52 SNF2.13XMYC::Kanmx* |
| YBC3019 | *MATa* | *leu2∆1(or ∆0) ura3-52(or ∆0) trp1∆63 his3∆200(or ∆1) rsc4∆::HIS3 [316.RSC4]* |
| YBC3020 | *MATa* | *leu2∆1(or ∆0) ura3-52(or ∆0) trp1∆63 his3∆200(or ∆1) rsc4∆::HIS3 ioc3∆::kanmx [316.RSC4]* |
| YBC3185 | *MATa* | *his3∆200 leu2∆1 lys2∆0 trp1∆63 ura3∆0(or -52) rsc1∆::HIS3 rsc2∆::LEU2 sir3∆::Natmx [316.RSC1]* |
| YBC3227 | *MATα* | *his3∆200 leu2∆1 lys2∆0 trp1∆63 ura3∆0(-52) rsc1∆::HIS3 rsc2∆::LEU2 isw1∆::kanmx* |
| YBC3228 | *MATα* | *his3∆1(or200) leu2∆0(or∆1) trp1∆63 ura3∆0(-52) rsc1∆::HIS3 rsc2∆::LEU2 ioc3∆::KanMX* [*314.rsc2.V457M*] |
| YBC3386 | *MATa* | *trp1∆63 his3∆200 ura3-52 leu2::PET56 GAL1prom-UBR1::HIS3 sth1::CUP1prom-STH1td::URA3* |
| YBC3387 | *MATa* | *trp1∆63 his3∆200 ura3-52 leu2::PET56 ubr1∆ sth1::CUP1prom-STH1td::URA3* |
| YBC3494 | *MATa* | *ade2-101 his3∆200 leu2∆1 trp1∆63 ura3-52 rsc2∆::Kanmx gcn5∆::HIS3* [*316.RSC2*] |
| YBC3495 | *MATa* | *ade2-101 his3∆200 leu2∆1 lys2-801 trp1∆63 ura3-52 rsc2∆::Kanmx gcn5∆::HIS3 isw1∆::Natmx* [*316.RSC2*] |
| YBC3496 | *MATa* | *ade2-101 his3∆200 leu2∆1 lys2-801 trp1∆63 ura3-52 rsc2∆::Kanmx gcn5∆::HIS3 isw1∆::Natmx [316.RSC2]* |
