# Supplementary material for: The chromatin remodelers RSC and ISW1 display functional and chromatin-based promoter antagonism
Source: eLife. 2015 Mar 30;4:e06073. doi: 10.7554/eLife.06073 (PMC4423118; doi:10.7554/eLife.06073)
Supplement: Supplementary file 2. — Table of plasmids. List of plasmid names and their sources used in this study. DOI: http://dx.doi.org/10.7554/eLife.06073.020 [file elife06073s002.docx]

# Supplemental Table 2. Plasmids used in this study.

| Number | Plasmid Name | Source |
| --- | --- | --- |
| 6 | pRS314 | a |
| 8 | pRS316 | a |
| 137 | p316.RSC7 | b |
| 604 | p314.RSC2.2XHA | c |
| 609 | p314.RSC1.3XMYC | c |
| 776 | p314.RSC2.V457M.2XHA | d |
| 777 | p314.RSC2.D461G.2XHA | d |
| 1060 | p314.RSC4 | e |
| 1083 | p314.rsc4-2 | e |
| 1211 | p416.ISW1.flag | f |
| 1212 | p416.ISW1 K227A.flag | f |
| 1411 | pWZ414-F13-H3.H4 WT | g |
| 1525 | p314.RSC1.F300S.3XMYC | This work |
| 1526 | p314.RSC1.Y297H.3XMYC | This work |
| 1527 | p314.RSC1.V417M.3XMYC | This work |
| 1528 | p314.RSC1.D421G.3XMYC | This work |
| 1696 | 414.H3-A7T.H4 | This work |
| 1697 | p414.H3-G33V.H4 | This work |
| 1698 | p414.H3-T6I.H4 | This work |
| 1701 | p414.H3-A7V.H4 | This work |
| 1702 | p414.H3-H4.R17C,H18Y | This work |
| 2301 | pWZ414-F13-K79A | h |
| 2302 | pWZ414-F13-K79Q | h |
| 3018 | p416.Met25.STH1 | This work |
| 3051 | p306.Sth1td | This work |
| a. Sikorski, R. S. and P. Hieter (1989). Genetics 122(1): 19-27. | | |
| b. Wilson B., et al (2006). Genetics 172 (2): 795-809 | | |
| c. Cairns, B. R., et al. (1999). Mol Cell 4(5): 715-723. | | |
| d. Schlichter, A. and B. R. Cairns (2005). Embo J 24(6): 1222-1231. | | |
| e. Kasten, M., et al. (2004). EMBO J 23(6): 1348-1359. | | |
| f. Tsuchiyama et al., 1999. Genes and Dev. 13-6 pp 686-697 | | |
| g. Zhang, W., et al. (1998). Embo J 17(11): 3155-3167. | | |
| h. Ng, H. H., et al. (2002). Genes Dev 16(12): 1518-1527. | | |
